# Sequential rTMS for MDD and OCD in a patient with left parietal perinatal ischemic infarct: a case report

**DOI:** 10.3389/fpsyt.2026.1899176

**Published:** 2026-08-26

**Authors:** Mark Odron, Vipul Reddy, Krrishika Saxena, Pailey DeBorde, Athreykrishna Parla, Charles Vigilia, Nisha Thunga, Jayleen Lu, Kenneth Blum, Rajendra D. Badgaiyan, Keerthy Sunder

**Affiliations:** 1 Division of Clinical Neuromodulation Research, Karma TMS, Palm Springs, CA, United States; 2 University of California, Berkeley, Berkeley, United States; 3 University of California, Riverside, Riverside, United States; 4 Sunder Foundation, Palm Springs, CA, United States; 5 Division of Addiction Research and Education, Center for Sports and Mental Health, Western University of Health Sciences, Pomona, CA, United States; 6 Department of Psychiatry, University of Vermont, Burlington, VT, United States; 7 Institute of Psychology, Eotvos Loránd University, Budapest, Hungary; 8 Department of Psychiatry, Wright University Boonshoft School of Medicine, Dayton, OH, United States; 9 Centre for Genomics and Applied Gene Technology, Institute of Integrative Omics and Applied Biotechnology, Nonakuri, Purba Medinipur, West Bengal, India; 10 Department of Psychiatry, Stanford University School of Medicine, Palo Alto, CA, United States; 11 Department of Psychiatry, Mt. Sinai University School of Medicine, New York City, NY, United States; 12 Department of Psychiatry, University of California, Riverside School of Medicine, Riverside, Riverside, CA, United States

**Keywords:** dorsolateral prefrontal cortex (DLPFC), major depressive disorder (MDD), neuromodulation, obsessive-compulsive disorder (OCD), repetitive transcranial magnetic stimulation (rTMS), yale-brown obsessive-compulsive scale (Y-BOCS)

## Abstract

Major depressive disorder (MDD) commonly co-occurs with obsessive-compulsive disorder (OCD), resulting in greater symptom severity, functional impairment, and suboptimal response to standard pharmacologic and psychotherapeutic interventions. These challenges underscore the need for neuromodulation strategies to target distinct neural networks implicated in mood regulation and compulsivity. This report describes outcomes of high-frequency (HF) repetitive transcranial magnetic stimulation (rTMS) of the left dorsolateral prefrontal cortex (DLPFC), and low-frequency (LF) rTMS of the right orbitofrontal cortex (OFC), in a patient with comorbid MDD, OCD, and a left parietal perinatal ischemic infarct. Over the course of treatment, serial psychometric assessments demonstrated progressive reductions in frequency and intensity of depressive symptoms, anxiety, and obsessive-compulsive behaviors, measured via Patient Health Questionnaire-9 (PHQ-9), Generalized Anxiety Disorder 7-Item Scale (GAD-7), and Yale-Brown Obsessive-Compulsive Scale (Y-BOCS). This case adds to the growing body of literature demonstrating efficacy of DLPFC and OFC stimulation for depression and OCD. Further large-scale, blinded, and randomized trials are warranted to examine the efficacy of sequential DLPFC and OFC stimulation for comorbid MDD and OCD compared with single-site DLPFC stimulation.

## 1 Introduction

The comorbidity of MDD and OCD has been associated with greater symptom severity, functional impairment, and increased suicidality (1–3). Additionally, studies have shown that patients with OCD comorbid with other mood disorders have poorer treatment outcomes compared to those with OCD alone (4). Repetitive transcranial magnetic stimulation (rTMS) is a non-invasive therapy that has demonstrated efficacy in treatment-resistant depression and various psychiatric conditions, including OCD (5, 33). High-frequency (HF) stimulation of the left dorsolateral prefrontal cortex (DLPFC) is an established approach for MDD, and has been shown to improve symptoms through modulation of prefrontal networks involved in executive function and emotional regulation (6, 7). In contrast, OCD has been associated with hyperactivity of cortico-striatal-thalamo-cortical (CSTC) circuits, including the orbitofrontal cortex (OFC). Inhibitory low-frequency (LF) stimulation of these regions has demonstrated potential benefit in decreasing compulsive symptoms (8, 9). Because MDD and OCD arise from distinct neural circuits, neuromodulation strategies focusing on each circuit may offer therapeutic advantages in patients with both conditions (5, 8). Additionally, structural brain lesions, such as parietal lobe ischemic infarcts, can lead to long-term alterations in cortical connectivity; focal lesions involving parietal and other cortical regions have been implicated in the emergence of OCD symptoms over time (10, 11). In this report, we describe the clinical course of a patient with recurrent MDD and OCD, whose psychiatric symptoms may be influenced by a perinatal parietal stroke. The patient underwent sequential dual-site rTMS, namely HF rTMS of the left DLPFC and LF rTMS of the right OFC. This case builds on prior studies of DLPFC and OFC stimulation in MDD and OCD (27).

## 2 Case presentation

**Figure 1:**
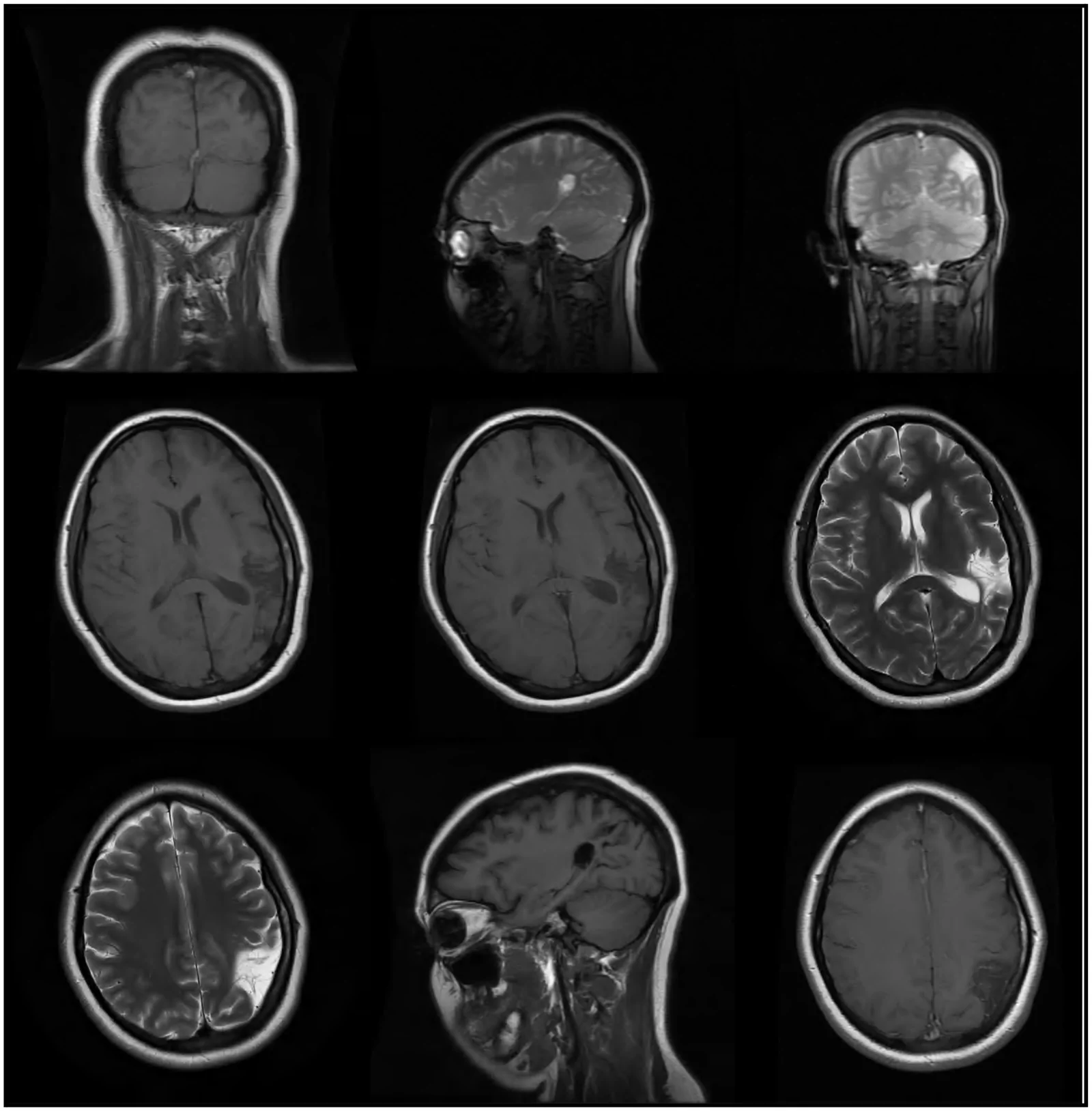
Left parietal lobe encephalomalacia consistent with an old MCA infarct.

Patient is a 26-year-old female with recurrent severe MDD and OCD, along with symptoms consistent with a diagnosis of generalized anxiety disorder (GAD). She reported a lifelong pattern of depression and anxiety beginning at approximately age 8, with exacerbations associated with psychosocial stressors, interpersonal conflicts, and emotional abuse during adolescence. Her mother reported onset of repetitive behaviors in the patient starting at approximately age 5. These behaviors involved evenness compulsions (e.g. spinning in place and knocking an even number of times, stubbing toes in both feet) and contamination compulsions (e.g. avoiding doorknobs, compulsive handwashing). These symptoms resulted in functional impairment across home, educational, and occupational settings. She began antidepressant treatment at age 22, but continued to experience significant symptoms such as anhedonia, concentration deficits, guilt, and psychomotor slowing. The patient also reported periods of passive self-harm thoughts without plan or intent, which she described as obsessions without compulsions. Past medical history was notable for a perinatal cerebrovascular accident (CVA) associated with hypoxic-ischemic injury of the left parietal lobe due to maternal prolonged labor. This resulted in recurrent seizures in the first 2 months of the patient’s life, with no seizure activity thereafter. Magnetic resonance imaging (MRI) in 2026 revealed a wedge-shaped area of encephalomalacia with surrounding gliosis measuring approximately 3.0 x 2.5 x 2.4 cm in the left parietal area, consistent with a remote infarct of the middle cerebral artery (MCA) territory (**Figure 1**). There was no evidence of acute ischemia, hemorrhage, or mass lesion. rTMS was therefore recommended to alleviate her symptoms, decrease functional impairment, and improve overall quality of life.

## 3 Methods

### 3.1 Subject

The subject was selected based on a diagnosis of recurrent MDD comorbid with OCD. Prior to treatment initiation, she was screened for contraindications to TMS, including ferromagnetic implants within 12 inches of the head, cardiac pacemaker, implantable cardioverter defibrillator, and history of active seizures and brain lesions (12). The subject had a remote history of perinatal CVA attributed to maternal prolonged labor during her delivery. Medical evaluation revealed no progressive neurologic deficits, and no history of seizures beyond infancy. A recent brain MRI demonstrated stable chronic ischemic changes in the left parietal lobe, with no evidence of acute pathology, and anatomically distinct from stimulation targets. Given the remote, non-progressive nature of her injury, along with absence of seizure activity for over 25 years, the patient was deemed appropriate for rTMS treatment in accordance with published safety recommendations (13). The treatment course consisted of an initial 36 sessions administered over approximately 7 weeks, followed by 10 additional sessions to consolidate gains, resulting in a total of 46 sessions over 10 weeks. The recommendation was based on clinical data demonstrating sustained durability of symptom improvements in those who underwent the recommended number of treatments (14, 32). The subject had been taking venlafaxine extended-release 300 mg daily at a stable dose for 12 months before initiating rTMS, with no changes in dosage or frequency throughout the TMS course. She had also participated in cognitive behavioral therapy (CBT) once every 2 weeks for 8 months before rTMS and continued at the same frequency throughout the TMS course.

### 3.2 Repetitive transcranial magnetic stimulation treatment

**Figure 2:**
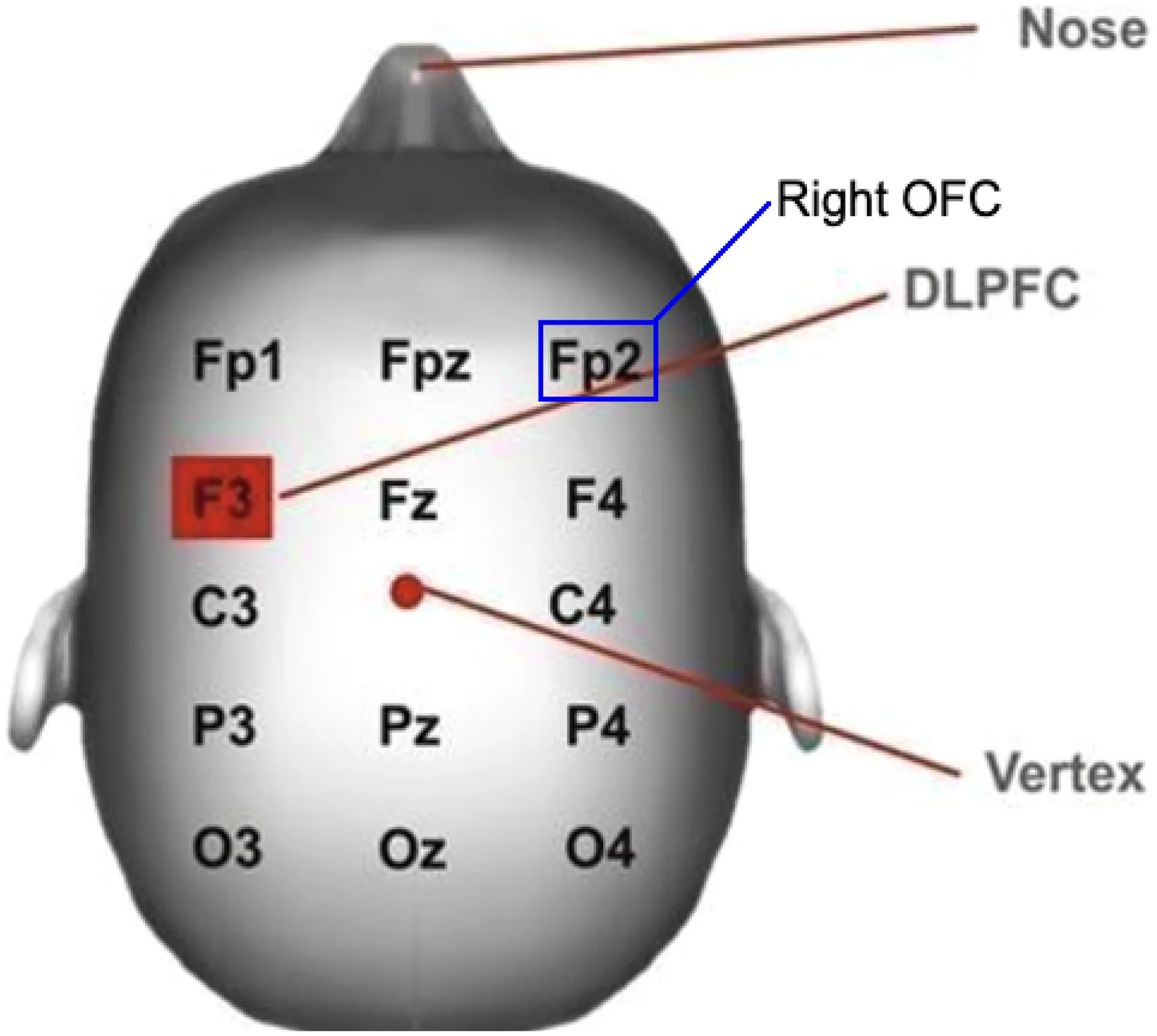
10–20 system.

Left-sided prefrontal stimulation was done using a conventional high-frequency rTMS protocol consistent with previously published methods from our group (30, 31). rTMS treatments were administered by certified clinical neurotechnologists using the Apollo TMS Therapy System. Using the International 10–20 system (**Figure 2**), the left DLPFC was localized, corresponding to the F3 location on the scalp (15). The resting motor threshold (RMT) was established as the minimum stimulation intensity over the primary motor cortex needed to produce a visible contralateral thumb twitch. Stimulation was delivered at 10 Hz with a total of 3000 pulses per session, organized into 75 trains of 40 pulses each, and separated by 11-second intertrain intervals. The subject’s RMT was determined to be 61%, and stimulation intensity was capped at 100% of this RMT. The patient was treated for a total of 46 sessions over 10 weeks. Tolerability was assessed through clinical observation and direct questioning regarding common adverse effects such as headache, scalp discomfort, neck pain, toothache, dizziness, and neurologic symptoms, before and after each session (12). No adverse effects were reported or observed.

Additionally, LF rTMS targeting the right OFC was added from session 19 onward to address persistent obsessive-compulsive symptoms. The right OFC was approximated at the right frontopolar area (Fp2) (15). Inhibitory stimulation (1 Hz) was delivered at this location for a total of 1200 pulses over 20 minutes. This protocol was selected based on prior studies demonstrating reductions in obsessive-compulsive symptoms following LF stimulation of hyperactive regions in CSTC circuits implicated in OCD (8, 9). Right OFC stimulation was administered sequentially following the standard left DLPFC treatment during each visit. All treatments were completed without any adverse events. The stimulation targets and parameters were selected according to the patient’s MDD and OCD diagnoses, and were not modified based on the remote parietal infarct. Initial left DLPFC stimulation alone was administered primarily for MDD while assessing for a potential secondary effect on OCD symptoms (36). Because the Y-BOCS score remained within the severe range by the midpoint assessment, right OFC stimulation was added from session 19 onward as an evidence-informed, response-guided augmentation.

### 3.3 Psychometric questionnaire administration

Symptoms were monitored using standardized psychometric questionnaires. The Patient Health Questionnaire-9 (PHQ-9), Generalized Anxiety Disorder-7 (GAD-7), and Yale-Brown Obsessive-Compulsive Scale (Y-BOCS) were administered as validated measures of depression, anxiety, and obsessive-compulsive symptom severity, respectively (16, 17). The PHQ-9 and GAD-7 were administered weekly throughout the treatment course, while the Y-BOCS was administered during the first week of treatment, at midpoint (session 19), and at the 36th and 46th (final) treatment sessions.

## 4 Discussion

**Figure 3:**
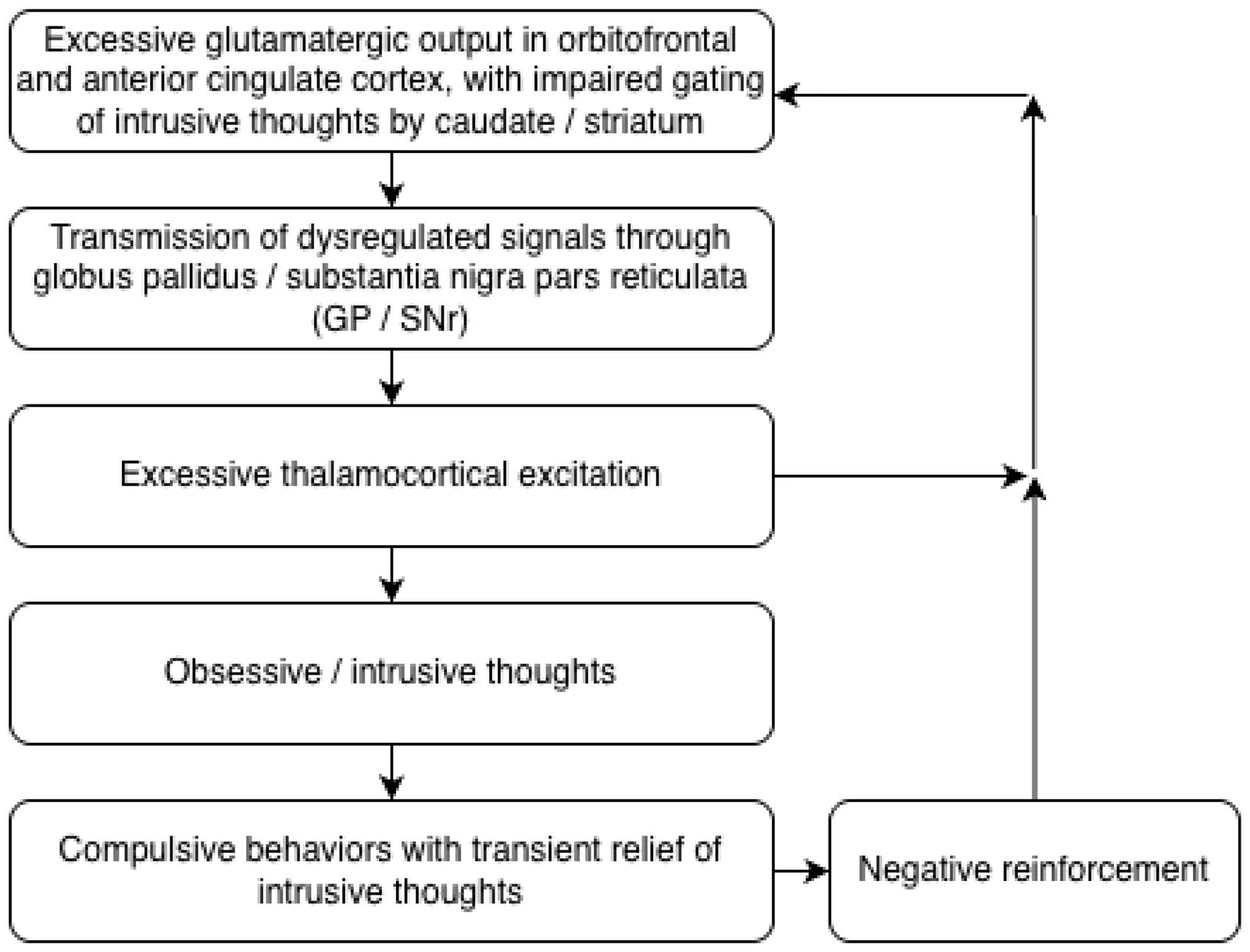
CSTC circuitry implicated in OCD.

Neurobiologic models of MDD implicate hypoactivity of the left DLPFC, an area involved in executive function, emotional regulation, and cognitive processing (18, 19). HF stimulation of this region is thought to increase cortical excitability and enhance top-down control over limbic structures involved in emotional processing. In contrast, OCD has been associated with hyperactivity within CSTC circuits, particularly involving the OFC and basal ganglia (20, 21). Excessive glutamatergic drive in the OFC and dorsal anterior cingulate cortex (dACC), along with impaired ability of the striatum to gate intrusive thoughts, are thought to contribute to recurrent excitation through the globus pallidus (GP), substantia nigra pars reticulata (SNr), and thalamocortical pathways which reinforce obsessive-compulsive behaviors (**Figure 3**) (8, 9, 21). Additionally, prior research, including functional MRI (fMRI) studies, revealed abnormal activation of the OFC during symptom provocation, reversal learning, and no-go tasks, along with decreased activation in the OFC after symptom improvement (35). Neuromodulation protocols targeting these distinct regions may therefore provide benefit in patients with concurrent mood and compulsive symptoms. Several studies have demonstrated that LF rTMS of the OFC can decrease the intensity and frequency of compulsive symptoms via modulation of hyperactive CSTC circuits (8, 9).

The presence of a remote perinatal infarct involving the left parietal lobe provides additional context for the patient’s neuropsychiatric symptoms. Early-life cerebrovascular injury can result in long-term cortical reorganization and altered connectivity across neural networks involved in cognitive control and emotional regulation (22–24). Although the parietal cortex is not typically considered a primary locus in mood and OCD-related conditions, it plays an important role in large-scale association networks that interact with prefrontal and limbic cortices. Network-based models from neuroimaging studies suggest that abnormalities in OCD extend beyond classical CSTC loops to include the dACC, DLPFC, and parietal regions involved in cognitive control and working memory (28). Functional connectivity analyses have demonstrated increased dACC modulation of parietal and prefrontal regions in patients with OCD compared with healthy controls, supporting the concept of distributed network dysfunction rather than strictly a CSTC disorder (28, 29). Additionally, studies of pediatric stroke survivors have shown increased rates of internalizing psychiatric symptoms, including depression and anxiety, highlighting the long-term neuropsychiatric consequences of early cerebrovascular injury (25). Reviews of perinatal stroke syndromes similarly emphasize that early vascular injury to the developing brain can cause persistent neurodevelopmental sequelae extending beyond sensorimotor deficits (22, 26). We hypothesize that early parietal ischemic injury may have altered the development of distributed cognitive-control networks, potentially increasing vulnerability to mood and obsessive-compulsive symptoms. In this context, sequential rTMS of prefrontal and orbitofrontal regions may represent a targeted approach for modulating networks involved in mood dysregulation and compulsivity. However, because functional connectivity imaging was not performed in this patient, these interpretations remain speculative.

**Figure 4:**
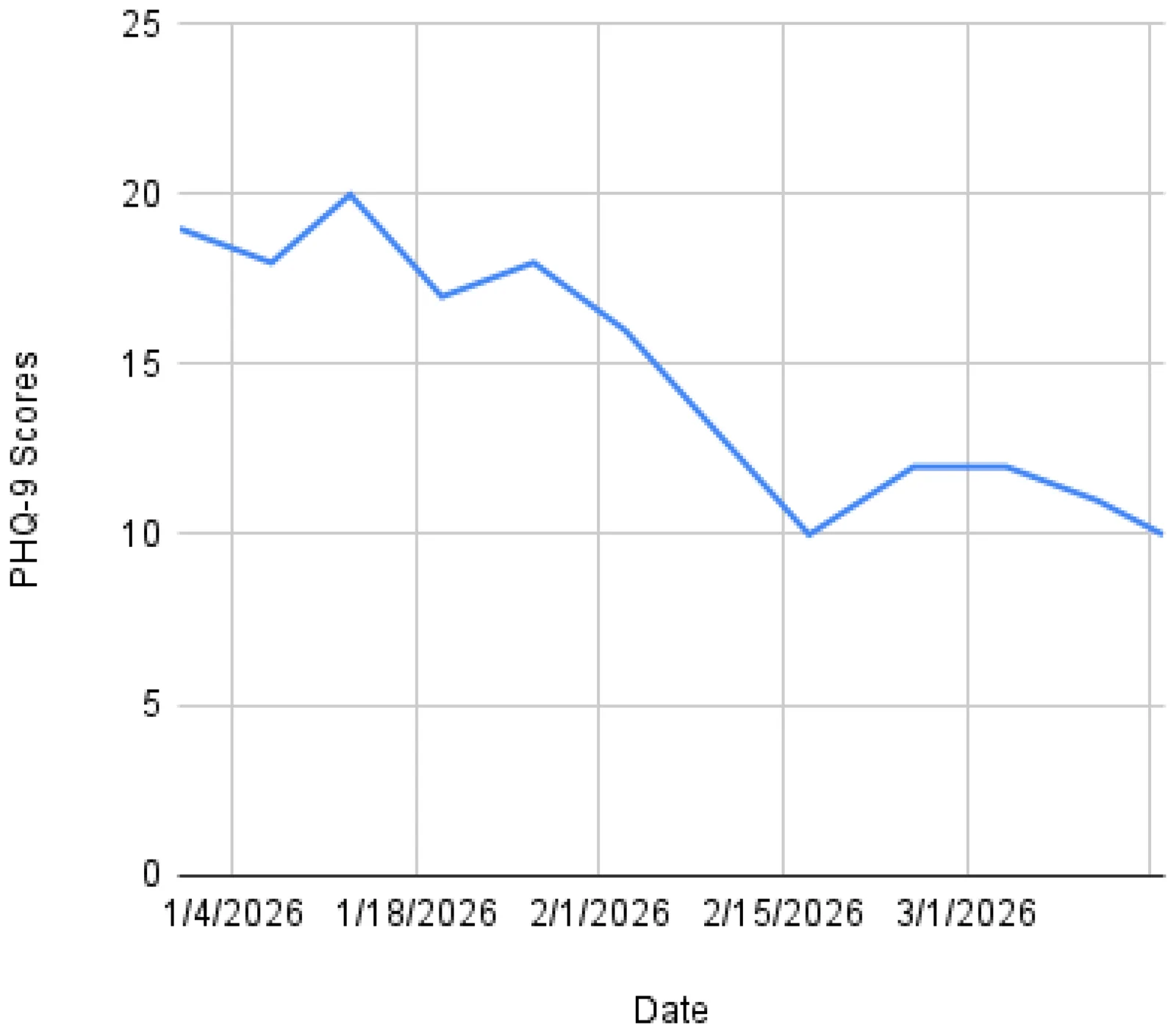
PHQ-9 scores.

**Figure 5:**
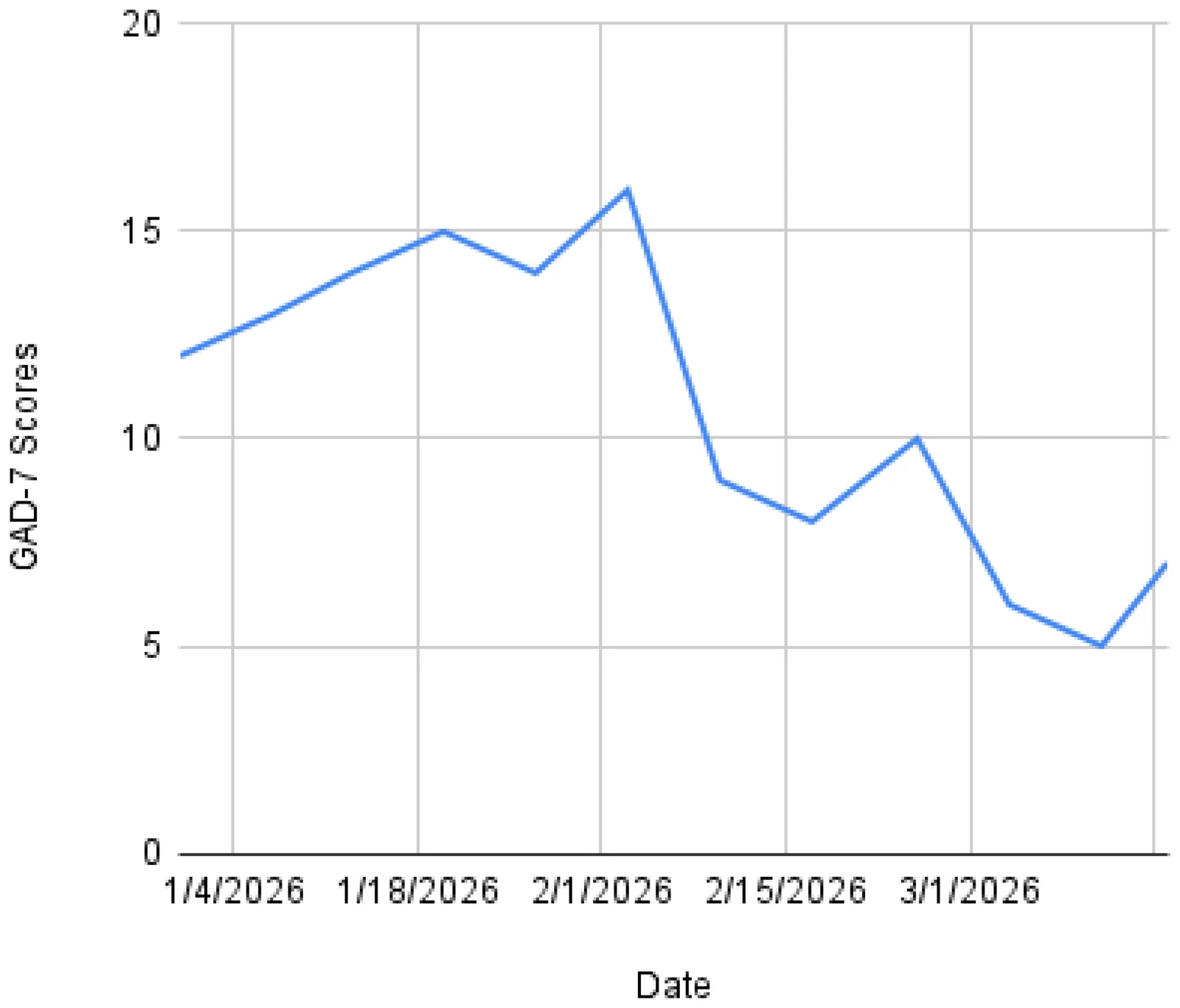
GAD-7 scores.

This case demonstrates gradual improvements in intensity and frequency of depressive symptoms, anxiety, and OCD following sequential rTMS of the left DLPFC and right OFC. Over the treatment course, the patient’s PHQ-9 scores decreased from 19 to 10 (47.4%), signifying a change from moderately severe to moderate MDD, while GAD-7 scores decreased from 12 to 7 (41.7%), signifying a change from moderate to mild anxiety (**Table 1**; **Figures 4**, **5**). Y-BOCS scores decreased from 28 to 18 (35.7%), signifying a change from severe to moderate OCD (**Table 2**). Addition of right OFC stimulation from session 19 onward coincided with sustained decreases in OCD symptoms. The results are consistent with prior literature suggesting that inhibitory OFC stimulation may decrease compulsive symptoms beyond the effects of left DLPFC stimulation alone (27). Overall, the temporal relationship between sequential rTMS and gradual psychometric improvements underscores the need for further studies involving dual-site rTMS as a potential approach for comorbid MDD and OCD. Although deep TMS (dTMS) protocols targeting the dACC and medial prefrontal cortex have demonstrated efficacy in OCD (34), OFC stimulation via rTMS may also represent a potential strategy for modulating CSTC circuitry. While remission was not reached for depressive and obsessive-compulsive symptoms during the study duration, the findings suggest that modulation of distinct circuits associated with mood and compulsivity may contribute to meaningful improvements for comorbid MDD and OCD. Consistent with a broader strategy of multi-locus rTMS for comorbid conditions, a prior case series also reported symptom reductions following sequential rTMS targeting the left DLPFC and bilateral supplementary motor cortex (SMA) in patients with comorbid MDD and OCD (37).

**Table 1:** PHQ-9 and GAD-7 scores.

PHQ-9 and GAD-7 scores	12/31/2025	1/7/2026	1/13	1/20	1/27	2/3	2/10	2/17	2/25	3/4	3/11	3/16
PHQ-9	19	18	20	17	18	16	13	10	12	12	11	10
GAD-7	12	13	14	15	14	16	9	8	10	6	5	7

**Table 2:** Y-BOCS scores.

Y-BOCS	12/31/2025	1/27/2026	2/25/2026	3/16/2026
Raw Score	28	27	22	18
Breakdown	16 (Obsessions) 12 (Compulsions)	14 (Obsessions) 13 (Compulsions)	12 (Obsessions) 10 (Compulsions)	10 (Obsessions) 8 (Compulsions)
Interpretation	Severe OCD	Severe OCD	Moderate OCD	Moderate OCD

## 5 Limitations

The use of rTMS for MDD and OCD in the context of a remote parietal infarct was limited to a single case report. Its feasibility must be further explored using large-scale studies in order to validate findings. Further studies are also warranted to determine whether comparable outcomes occur in patients with comorbid MDD and OCD without structural brain injury. Additionally, the use of self-reported psychometric questionnaires is subject to response bias and placebo effects. Lastly, continued venlafaxine and CBT may have contributed independently or synergistically to the observed improvements. Therefore, the effects cannot be attributed solely to rTMS. However, this potential rTMS effect may be explored in future studies given the timing of symptom improvements after rTMS initiation.

## 6 Patient perspective

I’ve been suffering with depression, OCD, and anxiety since early childhood, along with a stroke at birth and seizures for the first 2 months of my life. I also suffered with selective mutism at school and throughout adolescence. I’ve had chronic passive self-harm thoughts, and difficulty concentrating due to persistent intrusive thoughts, such as obsessions with cleanliness and evenness. My mother noted OCD symptoms in my childhood, including rubbing my hands together, spinning in a circle a certain number of times and then reversing it, and excessive agitation when my belongings were “not just so.”

Weeks after I started the DLPFC and then the OFC protocol, I started feeling relief. I woke up one Saturday morning and opened my front door to see that the mountains in front of me had more saturated colors. I found that I could also hear more of the nature surrounding me because my mind was not racing as much as it had been. Over the following weeks, I found everyday life to be a little easier to get through. My boss even noted in one of our meetings that I had been more empathetic and focused. I am still working on a number of symptoms in therapy. However, TMS has been a huge help in relieving the overwhelming things that I’ve had for most of my life. I started playing games, cooking, and painting again in my free time. I’ve also been more communicative with friends and loved ones.

## 7 Conclusion

Neuromodulation strategies are relatively understudied for OCD with an identifiable organic pathology (e.g. stroke, traumatic brain injury, tumors). rTMS has shown efficacy for MDD, GAD, and OCD, which involve dysregulation of prefrontal and corticostriatal circuits. Conventional left DLPFC stimulation may not optimally address comorbid MDD and OCD, especially in the background of an identifiable organic etiology. In this context, sequential stimulation of the left DLPFC and right OFC may provide a framework for modulating circuits involved in mood regulation and compulsivity. Although an association cannot be drawn from a single case, the coincidence of psychometric improvements following rTMS warrants further investigation using large-scale, blinded, and randomized trials to evaluate the potential efficacy of dual-site rTMS for comorbid MDD and OCD.

## Data Availability

The original contributions presented in the study are included in the article/supplementary material. Further inquiries can be directed to the corresponding author.
